# Investigating the diagnostic value of quantitative parameters based on T2-weighted and contrast-enhanced MRI with psoas muscle and outer myometrium as internal references for differentiating uterine sarcomas from leiomyomas at 3T MRI

**DOI:** 10.1186/s40644-019-0206-8

**Published:** 2019-04-01

**Authors:** Mahrooz Malek, Maryam Rahmani, Seyyedeh Mahdieh Seyyed Ebrahimi, Elnaz Tabibian, Azadeh Alidoosti, Pariya Rahimifar, Setareh Akhavan, Ziba Gandomkar

**Affiliations:** 1 Department of Radiology, Medical Imaging Center, Imam Khomeini Hospital Complex (IKHC), Tehran University of Medical Sciences (TUMS), End of Keshavarz Blvd, Tehran, 1419733141 Iran; 2 Department of Obstetrics and Gynecology, Tehran University of Medical Sciences (TUMS), Tehran, Iran; 3 The University of Sydney, Discipline of Medical Imaging and Radiation Sciences, Image Optimisation and Perception Group (MIOPeG), Sydney, NSW Australia

**Keywords:** Contrast-enhanced MRI, Leiomyomas, Magnetic resonance imaging, T2 mapping, Uterine sarcomas

## Abstract

**Background:**

Post-hysterectomy histopathological examination is currently the main diagnostic tool for differentiating uterine sarcomas from leiomyomas. This study aimed to investigate the diagnostic accuracy of preoperative quantitative metrics based on T2-weighted sequences and contrast-enhanced MRI (CE-MRI) for distinguishing uterine sarcomas from leiomyomas.

**Materials and methods:**

The institutional review board approved the study. Sixty-five women confirmed to have a total of 105 lesions participated. Routine pelvic MRI sequences, T2 map and CE-MRI images were performed preoperatively using a 3 T MR scanner. Six quantitative metrics—T2 mapping parameter, T2 scaled ratio, tumor myometrium contrast ratio on T2, tumor psoas contrast ratio on T2, tumor myometrium contrast-enhanced ratio, and tumor psoas contrast-enhanced ratio—were extracted from the acquired image sets. Chi-square test was used to compare the percentage of malignant lesions with the central necrosis to the corresponding percentage for the benign masses. Using the area under receiver operating characteristic (AUC) curve, the performance of different metrics for distinguishing uterine sarcomas from leiomyomas was measured. Moreover, for each metric, we extracted the optimal cut-off value. The values of sensitivity, specificity, negative predictive value, and positive predictive value were calculted for the classifiers based on different metrics.

**Results:**

The average age, average lesion size, and proportion of premenopausal women in benign and malignant groups were comparable in our dataset. The signal intensity of uterine sarcomas at T2-weighted sequences was significantly higher than that of leiomyomas (*p* < 0.001), while intensity at T1-weighted sequences exhibited no significant difference between the two masses (*p* = 0.201). Our data also suggested that a central necrosis was ten times more common among malignant lesions compared to benign ones (*p* < 0.001). Among different metrics, T2 mapping parameter achieved the highest AUC value and accuracy in differentiating two groups. Three measures—T2 scaled ratio, tumor myometrium contrast ratio on T2, and tumor myometrium contrast-enhanced ratio—achieved a sensitivity of 100%, therefore none of the malignant lesions would have been missed if these metrics had been adopted in patient management.

**Conclusions:**

The findings suggested that the evaluated metrics could be useful in the preoperative assessment of myometrial masses to differentiate uterine sarcomas from leiomyomas. The proposed framework has major implications for improving current practice in the management of myometrial masses.

## Background

Leiomyomas are the most common uterine tumors affecting women of reproductive age. The prognosis is usually excellent with the appropriate selection of treatment regimens. On the other hand, uterine sarcomas are rare, accounting for less than 10% of uterine malignancies, and exhibit poor prognosis [1–5].

Uterine sarcomas are usually treated through hysterectomy followed by adjuvant chemotherapy, while uterine-preserving therapeutic procedures such as uterine arterial embolization [6] or gonadotropin-releasing hormone analogues [7] are available for most subtypes of leiomyomas. Therefore, accurate preoperative differentiation of uterine masses is of considerable value in the selection of optimal treatment, especially for patients of childbearing age.

Although some preoperative findings and the presence of certain risk factors support the possibility of uterine sarcoma, no specific clinical presentations differentiatie uterine sarcomas from leiomyomas [3]. Previous studies suggested that neither endometrial sampling [8, 9] nor pelvic imaging-guided uterine mass biopsy [3] could provide a sufficient amount of tissue for proper histopathological examination preoperatively. Moreover, the spillage of malignant cells during the biopsy procedure could increase the uterine sarcoma stage [10–13].

Magnetic resonance imaging (MRI) plays a prominent role in the preoperative assessment of uterine masses. Leiomyomas typically present as well-defined hyposignal lesions at T2-weighted sequences, while the intra-tumoral hypersignal at T1 or T2 is considered suspicious for uterine sarcomas [14, 15]. However, previous studies also showed that atypical leiomyomas and uterine sarcomas can have a similar appearance on routine MRI, and therefore might be indistinguishable from each other [14, 16–20]. Currently, due to this overlap and lack of uniform diagnostic criteria for differentiating uterine sarcomas from leiomyomas, most patients presenting borderline symptoms are treated aggressively with a hysterectomy although some might have been suitable for uterine-preserving treatment. More aggressive treatment strategies are adopted because under-interpretation of uterine sarcomas might delay necessary treatment and, hence, worsen the prognosis. Also, in 2014 the US Food and Drug Administration (FDA) issued tough warning against using laparoscopic power morcellators in hysterectomy or myomectomy due to the risk of spreading unsuspected cancerous tissue [21].

Despite the importance of preoperative differentiation of uterine sarcomas from leiomyomas, only a few previous studies focused on distinguishing uterine sarcomas from leiomyomas based on quantitative metrics extracted from the acquired MRI sequences. Namimoto et al. [22] used the signal intensity on T2-weighted images combined with diffusion-weighted imaging to differentiate uterine sarcomas from benign leiomyomas and indicated that the mean tumor–myometrium contrast ratio of sarcomas was significantly higher than that of the leiomyomas. However, there was considerable overlap between the benign and malignant groups. Therefore, these parameters were not sufficient for a reliable differentiation of uterine sarcomas from leiomyomas and post hysterectomy histopathological examination remains the mainstay of definite differentiation.

Recent studies utilized quantitative T2-based parameters such as the T2 mapping parameter in evaluating ovarian [23] and breast tumors [24]. Moreover, Kang et al. indicated that the scaled signal intensity of uterine fibroids on T2-weighted image could be a useful indicator for predicting patients’ responses to uterine embolization treatment [25]. To the best of our knowledge, however, the quantitative T2-based parameters have not been used to differentiate uterine masses from leiomyomas. Central necrotic areas in contrast enhanced MRI (CE-MRI) have been considered helpful in differentiating uterine body masses. In this study, we evaluated the efficacy of quantitative T2-based MRI parameters, presence of central necrosis on CE-MRI images, and quantitative parameters from CE-MRI in differentiating uterine body sarcomas from leiomyomas.

## Materials and methods

### Patients

Our institutional review board approved the study. All patients in our hospital who had myometrial mass on ultrasound exam from March 2016 to June 2017 and were candidates for total hysterectomy (open surgery or laparoscopic) or myometrial tumor resection according to gynecological criteria were included in the study. Informed consent forms were obtained from all participants. Women with any electronic implant or device such as a pacemaker, neuron-stimulator, inner ear prosthesis, or insulin pump were excluded. Patients were also excluded if the time between MRI and surgery was more than 6 weeks. In total, 65 women confirmed to have a total of 105 lesions with an average age of 42.1 ± 11.7 were recruited for the study. All included patients underwent hysterectomy and weighed less than 100 kg. Information regarding patients’ menopausal status was collected during the subject recruitment process. The size of lesions was also recorded. After the hysterectomy, the final diagnosis for each lesion was made based on consensus of experienced pathologists.

### Imaging protocol

All patients underwent the standard MRI protocol for the assessment of uterine masses using a 3-Tesla MR imager (Magnetom Trio, Siemens, Erlangen, Germany). Briefly, the subject was asked to fast three hours prior to the examination. Hyoscine butylbromide, which is an intra-muscular anti-peristaltic agent, was administered prior to initiation of the scan. All subjects were positioned supine on the MR scanner table with the 4 channel phased-array coil positioned over the pelvis. Routine sequences for uterine masses were acquired for each patient. After that, for all patients, gadolinium contrast medium at 0.2 mmol/kg dose (Dotarem, Guerbet, Germany) was administered to produce CE-MRI at equilibrium phase (about 120 to 180 s after contrast injection).

### Image analysis

T2 signal (high or low in comparison by outer myometrium), T1 hyperintensity foci, and six quantitative metrics (T2 mapping parameter, T2 scaled ratio, tumor myometrium contrast ratio on T2, tumor psoas contrast ratio on T2, tumor myometrium contrast enhanced ratio, and tumor psoas contrast enhanced ratio) were calculated from the acquired sequences. All quantitative metrics were calculated off-line using PACS system by a radiologist with eight years of experience in gynecology oncology imaging.

To calculate the T2 mapping parameter, two-dimensional multiecho (six TE values from 10 to 61 ms) spin-echo sequence was utilized. The multiecho sequence was thresholded so that the noisy pixels were removed. The thresholding step was followed by fitting a mono-exponential decay function to each pixel to generate the T2 mapping parameter (briefly, T2 map).

For T2 scaled ratio calculation, the region of interest (ROI), which outlined the entire tumor while avoiding healthy tissue, was defined by an experienced radiologist. The ROIs (50 to 150 mm^2^) that encompassed the rectus abdominis muscle and the subcutaneous fat layer were located on the image by the radiologist, who ensured that the surrounding structures were excluded from the ROI. The radiologist also ensured that the ROIs did not contain hemorrhage and calcifications, which would affect both T1 and T2 signals. Scaled signal intensity ratio (or briefly T2 scaled ratio) was then calculated using (1) [26]. T2 scaled ratio ranged from 0 to 1 with 1 indicating the intensity of fat and 0 representing the intensity of the rectus abdominis. To ensure that variations in ROI selection did not have a detrimental effect on the measure, the procedure was performed three times and the average value was reported.

1 $$ T2\  Scaled\ Ratio=\frac{\mathrm{Signal}\ \mathrm{intensity}\ \mathrm{of}\ {\mathrm{ROI}}_{Mass}-\mathrm{Signal}\ \mathrm{intensity}\ \mathrm{of}\ {\mathrm{ROI}}_{\mathrm{Rectus}}}{\mathrm{Signal}\ \mathrm{intensity}\ \mathrm{of}\ {\mathrm{ROI}}_{Fat}-\mathrm{Signal}\ \mathrm{intensity}\ \mathrm{of}\ {\mathrm{ROI}}_{\mathrm{Rectus}}} $$

To calculate tumor myometrium contrast ratio on T2, the largest possible ROI was placed over the mass, while cystic or necrotic areas, large vessels, calcification, and hemorrhage were avoided. An ROI that included the normal outer myometrium was also defined and tumor myometrium contrast ratio on T2 was calculated using (2).

2 $$ \mathrm{TumorMyometriumContrastRatioonT}2=\frac{\mathrm{Signal}\ \mathrm{intensity}\ \mathrm{of}\ {\mathrm{ROI}}_{Mass}-\mathrm{Signal}\ \mathrm{intensity}\ \mathrm{of}\ {\mathrm{ROI}}_{\mathrm{outer}\ \mathrm{myometrium}}}{\mathrm{Signal}\ \mathrm{intensity}\ \mathrm{of}\ {\mathrm{ROI}}_{\mathrm{outer}\ \mathrm{myometrium}}} $$

Previously it was shown that presentation of the outer myometrial layer could change during the menstrual cycle [27]. To overcome this effect, as shown in (3), we also calculated tumor psoas contrast ratio and assumed psoas muscle as an internal reference.

3 $$ \mathrm{TumorMyometriumContrastRatioonT}2=\frac{\mathrm{Signal}\ \mathrm{intensity}\ \mathrm{of}\ {\mathrm{ROI}}_{Mass}-\mathrm{Signal}\ \mathrm{intensity}\ \mathrm{of}\ {\mathrm{ROI}}_{\mathrm{psoas}\ \mathrm{muscle}}}{\mathrm{Signal}\ \mathrm{intensity}\ \mathrm{of}\ {\mathrm{ROI}}_{\mathrm{psoas}\ \mathrm{muscle}}} $$

We also calculated the tumor psoas and tumor myometrium contrast ratios on CE-MRI image at the equilibrium phase (about 120 to 180 s after contrast injection). Similar metrics have been previously utilized to assess the depth of myometrial invasion in endometrial cancer [28]. The presence of the central necrosis was assessed on CE-MRI by an experienced radiologist. Only central pocket-like areas with well-defined boundaries were considered, as the scattered areas of necrosis do not have diagnostic value. The hypersignality on T1- and T2-weighted images were also qualitatively evaluated.

### Statistical analysis

The patients’ characteristics in the malignant cohort were compared to those in the benign cohort to assure that two groups are analogous. The patients’ age and lesion size were compared using two independent sample t-test while the proportion of the premenopausal women in each group was compared using Chi-Square test.

For evaluting the efficacy of signal intensity in distinguishing malignant lesions from benign ones, the percentages of hypersignal lesions on T1 and T2-weighted sequences were compared between benign and malignant groups using Chi-Square test. We also compared the percentage of malignant lesions with the central necrosis to the corresponding figure for the benign masses using Chi-Square test. The intensity of signal on T1 and T2 sequences and the presence of central necrosis were assessed visually by an experienced radiologist. To ensure that intra-observer reliability is acceptable, she was asked to evaluate these metrics twice after a wash-out period (more than 12 months later). The agreement between two readings were assessed using the Cohen’s Kappa coefficient, which was interpreted as suggested by McHugh [29].

The extracted quantitative metrics (i.e. T2 map, T2 scaled ratio, tumor myometrium contrast ratio on T2, tumor psoas contrast ratio on T2, tumor myometrium contrast enhanced ratio, and tumor psoas contrast enhanced ratio) were also compared between two groups using two independent sample t-test. The metrics that differed significantly between benign and malignant lesions were determined. All statistical analyses were performed using SPSS, version 22.0 (IBM Corp., Armonk, NY, USA).

The ultimate goal of extracting the quantitative metrics is utilizing them to differentiate benign masses from the malignant ones. For evaluating their effectiveness in this task, we generated Receiver Operating Characteristic (ROC) curve for each metric. The Area under ROC curve (AUC) was then calculated for each measure. AUC measures how well a metric can discriminate malignant lesions from the benign ones. The upper bound for AUC is 1 which suggests a perfect classifier while a value of 0.5 indicates a chance classifier. In addition, the optimal operational point (the knee point) was also extracted from each ROC curve. The knee point indicates the transition from rapid upsurge of sensitivity to rapid decrease of specificity. By applying the cut-off threshold corresponding to the knee point, the masses were either classified as benign or malignant. Five measures of accuracy including sensitivity, specificity, Negative Predictive Value (NPV), Positive Predictive Value (PPV), and overall accuracy were extracted for each metric.

## Results

Table 1 shows the characteristics of the 65 women with 105 uterine lesions who were included in this study. The percentages of malignant lesions and cases were 20 and 21.5%, respectively. The mean age of malignant and benign patients did not differ significantly (39.5 ± 11.2 versus 42.8 ± 13.3, *p* = 0.252). There was no significant difference between malignant and benign groups regarding mean lesion size and menopausal status.

**Table 1:** The patients’ characteristics

Parameters	Benign	Malignant	Overall	*p*-value
Number of patients (%)	51 (78.5%)	14 (21.5%)	65	–
Number of lesions (%)	84 (80%)	21 (20%)	105	–
Premenopausal women (%)	52 (61.9%)	13 (61.9%)	65 (61.9%)	1.00
Age(mean ± STD, range)	42.8 ± 13.3 (21–66)	39.5 ± 11.2 (18–68)	42.1 ± 11.7 (18–68)	0.25
Lesion size(mean ± STD, range)	68.2 ± 41.8 (8–219)	79.5 ± 49.5 (20–192)	70.5 ± 45.1 (8–219)	0.70

**Fig. 1:**
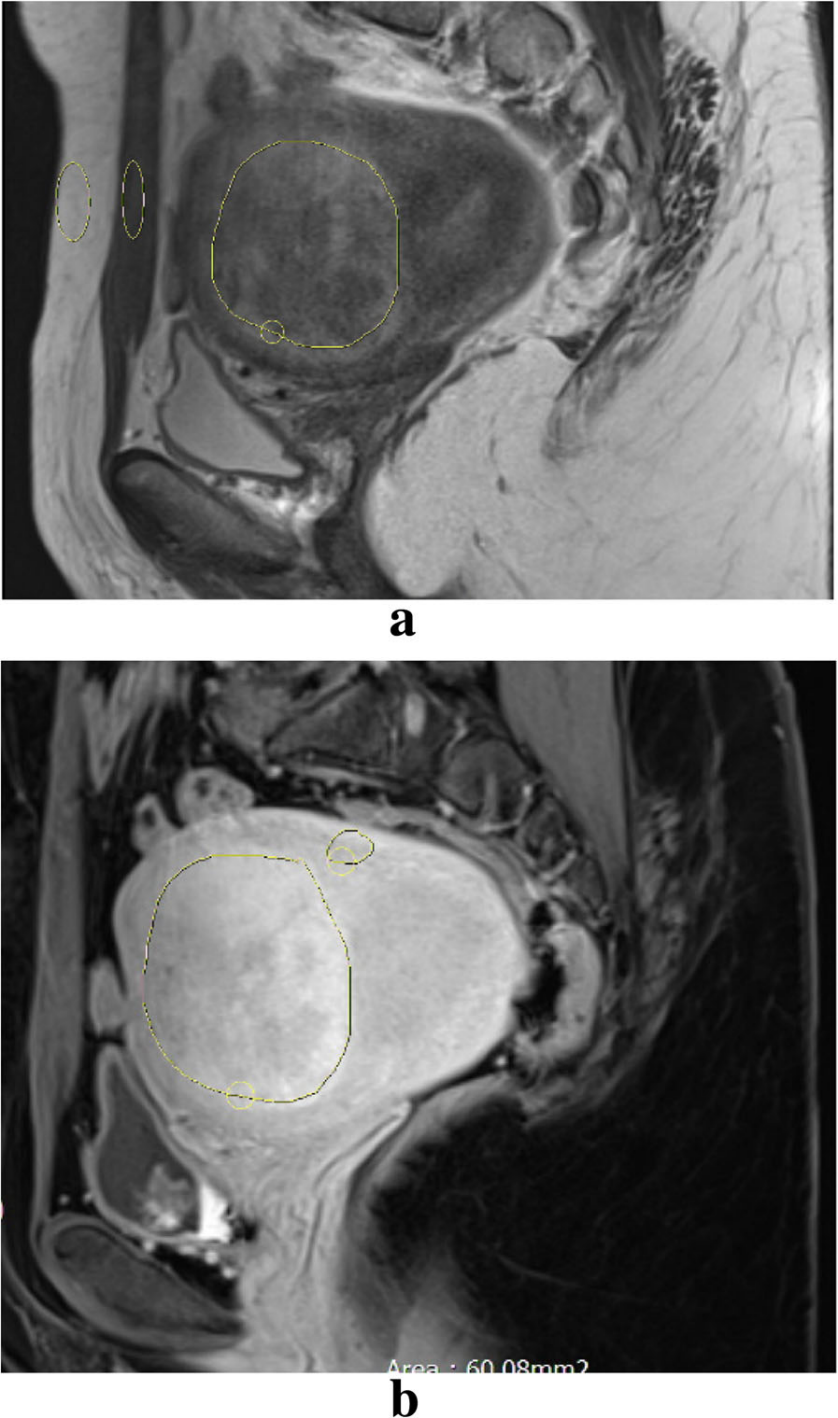
A 22-year-old patient with history of AUB and cellular leiomyoma on pathology. **a** A round high signal myometrial lesion on sagittal T2-weighted image, T2 scaled ratio = 0.28. **b** Sagittal contrast enhanced T1-weighted image with tumor psoas contrast enhanced ratio = 1.100

**Fig. 2:**
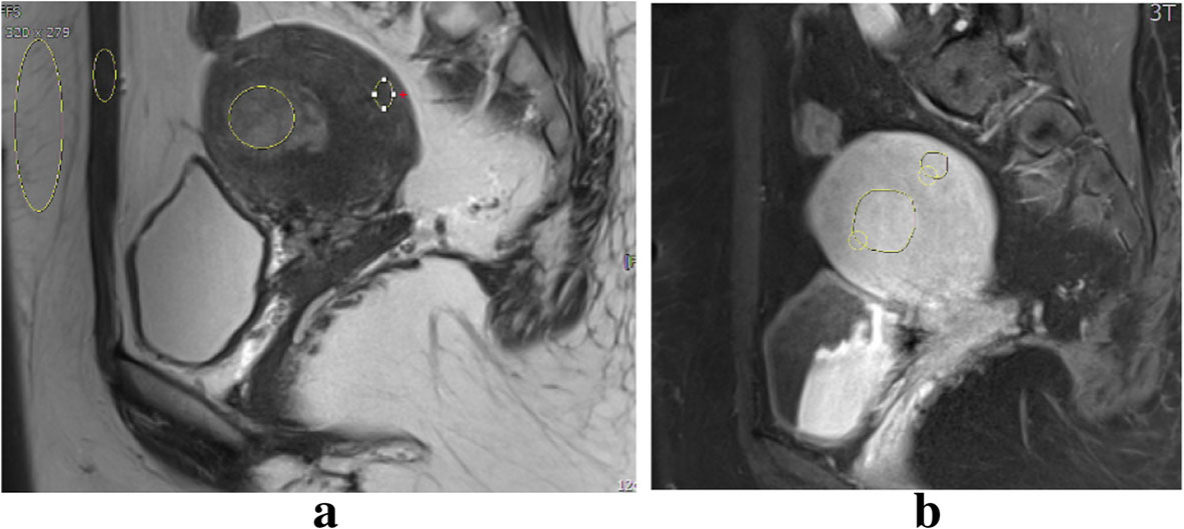
A 34-year-old patient with history of AUB and endometrial stromal sarcoma on pathology. **a** A round high signal myometrial lesion on sagittal T2-weighted image, tumor myometrial contrast ratio = 0.36, tumor psoas contrast ratio = 1.613. **b** Sagittal contrast enhanced T1-weighted image with tumor myometrial contrast enhanced ratio = 0.352 and tumor psoas contrast enhanced ratio = 1.482

The single intensity on T1 and T2 sequences was also compared between malignant and benign groups. The comparisons are summarized in Table 2. As shown, all malignant lesions included in the dataset showed high signal intensity on T2-weighted sequences whereas 70% of benign lesions presented as hyposignal on T2-weighted images (*p* < 0.001). At T1-weighted sequences, no significant difference between the intensities of the two groups was observed (*p* = 0.201). In addition, our data suggested that a central necrosis was ten times more common among malignant lesions than benign ones (p < 0.001). Figure 1 shows sagittal T2-weighted and contrast enhanced T1-weighted images for a 22-year-old patient with cellular leiomyoma while Fig. 2 indicates similar images for a 34-year-old patient endometrial stromal sarcoma. Assessing the intra-radiologist variability of metrics presented in Table 2 led to Cohen’s Kappa coffeicients above 0.91 for all three metrics, which is suggestive of almost perfect agreement between two readings and reliability of using these metrics.

**Table 2:** Comparison of quantitative variables between malignant and benign groups

Variable	Status	Benign (%)	Malignant (%)	Total (%)	*p*-value
Predominant signal on T2-weighted	Low	59 (70.2%)	0 (0%)	59 (56.2%)	< 0.001
	High	25 (29.8%)	21 (100%)	46 (43.8%)	
	Total	84 (100%)	21 (100%)	105 (100%)	
Hypersignality T1-weighted	No	78 (94%)	18 (85.7%)	96 (92.3%)	0.20
	Yes	5 (6%)	3 (14.3%)	8 (7.7%)	
	Total	83 (100%)	21 (100%)	104 (100%)	
Central Necrosis	No	80 (95.2%)	11 (52.4%)	91 (86.7%)	< 0.001
	Yes	4 (4.8%)	10 (47.6%)	14 (1.3%)	
	Total	84 (100%)	21 (100%)	105 (100%)

**Fig. 3:**
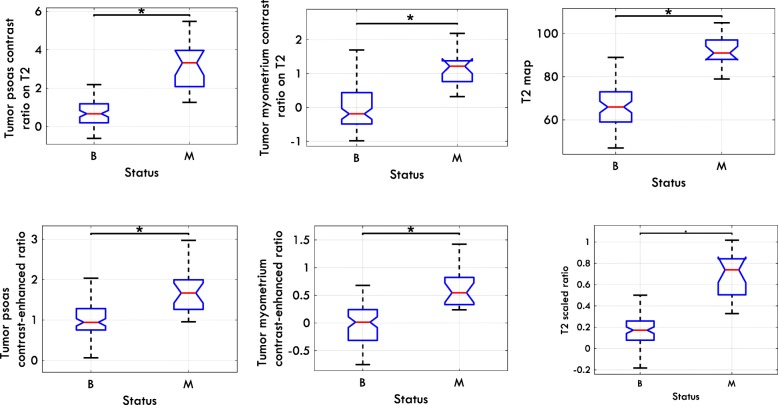
The distribution of extracted metrics among benign (B) and malignant (M) lesions. Each plot shows one of the T2 parameters. Significant difference between two groups is shown using asterisks (*)

Table 3 presents the values for mean and range of the extracted quantitative variables for the entire population, benign lesions, and malignant ones. The mean values of all variables differed significantly between benign and malignant groups (p < 0.001). The distribution of the variables is shown in Fig. 3. As shown in Fig. 3 and Table 3, all extracted variables were higher in the malignant group than in the group with benign lesions.

**Table 3:** The mean, standard deviation, 95% confidence interval, and the range of the extracted quantitative variables for the entire population along with the mean values of each parameter for benign and malignant lesions

Variable	Mean ± STD, (95% CI)	Range Min-Max	# Missing	Status	No.	Mean ± STD
T2 Map^a^	72.77 ± 15.06 (69.36 -75.74)	47–105	17	B	68	66.78 ± 10.94
				M	20	93.15 ± 7.14
T2 Scaled Ratio^a^	0.28 ± 0.26 (0.23 -0.33)	−0.18-1.02	1	B	84	0.19 ± 0.18
				M	20	0.66 ± 0.21
Tumor Myometrium Contrast Ratio on T2^a^	0.2 ± 0.75 (0.06 -0.35)	−0.99-2.19	4	B	82	−0.02 ± 0.61
				M	19	1.12 ± 0.55
Tumor Psoas Contrast Ratio on T2^a^	1.32 ± 1.42 (1.05 -1.58)	−0.61-5.48	0	B	84	0.85 ± 1.02
				M	21	3.19 ± 1.23
Tumor Myometrium Contrast Enhanced Ratio^a^	0.11 ± 0.45 (1.03 -1.23)	−0.75-1.49	4	B	82	0.00 ± 0.39
				M	19	0.61 ± 0.33
Tumor Psoas Contrast Enhanced Ratio^a^	1.14 ± 0.55 (0.02 -0.20)	−0.14-2.97	0	B	84	1.00 ± 0.44
				M	21	1.68 ± 0.58

^a^Indicates a variable that led to a p < 0.001; STD shows standard deviation; B and M represent benign and malignant groups respectively; 95% CI shows 95% confidence interval for the mean values and # Missing indicate number of missing cases; No. stands for number of cases in each group

### ROC analysis

**Fig. 4:**
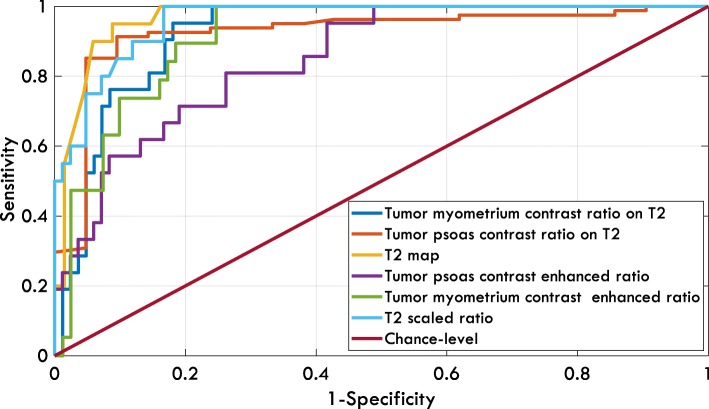
The ROC curves generated for three metrics

As all metrics resulted in a *p*-value< 0.001 (Table 3), they could be helpful in making the ultimate diagnosis about a uterine mass and distinguishing malignant from benign cases. To evaluate the diagnostic value of these metrics, ROC curves for each metric were generated. As explained in the Methods section, the values of AUC, sensitivity, specificity, NPV, PPV, and overall accuracy were calculated. The ROC curves are shown in Fig. 4. The results are shown in Table 4. The accuracy metrics for two variables, which led to a significant p-value in Table 2, were also computed and are presented in Table 4.

**Table 4:** Performances of different quantitative variables in distinguishing malignant lesions from benign ones

Variable (Cut-off Point)	AUC (95% Confidence Interval)	Sensitivity (%)	Specificity (%)	NPV (%)	PPV (%)	Accuracy (%)
Signal intensity of T2	–	100	70.2	100	45.7	76.2
Central Necrosis	–	47.6	95.2	87.9	71.4	85.7
T2 Map (82.5)	0.974 (0.930–0.988)	95	91.2	98.4	76.0	92.0
T2 Scaled Ratio (0.32)	0.952 (0.914–0.983)	100	83.3	100	58.8	86.5
Tumor Myometrium Contrast Ratio on T2 (0.31)	0.930 (0.851–0.966)	100	70.7	100	44.2	76.2
Tumor Psoas Contrast Ratio on T2 (1.58)	0.902 (0.787–0.974)	95.2	82.1	98.6	57.1	84.8
Tumor Myometrium Contrast Enhanced Ratio (0.23)	0.887 (0.835–0.968)	100	74.4	100	47.5	79.2
Tumor Psoas Contrast Enhanced Ratio (1.23)	0.840 (0.742–0.900)	81	73.8	93.9	43.6	75.2

The values of Area under Receiver operating Characteristics (AUC) curve, sensitivity, specificity, Negative Predictive Value (*NPV*), Positive Predictive Value (*PPV*) and accuracy are shown. The highest value in each column is shown in bold. The cut-off points corresponding to knee points of the receiver operating characteristics curves are also presented. The confidence interval for AUC was computed using bootstrap method

As shown, the T2 Map achieved the highest AUC, PPV, and overall accuracy. However, 5% of malignant cases would have been missed if the classifier based on this metric at its optimal operating point had been utilized. Alternatively, the classifiers based on signal intensity of T2, T2 scaled ratio, tumor myometrium contrast ratio on T2, or tumor myometrium contrast enhanced ratio obtained a sensitivity of 100%, which indicates none of the malignancies would have been misclassified as a benign mass if any of these metrics had been adopted. Among these four metrics, T2 scaled ratio obtained the highest specificity and overall accuracy, suggesting that, at the expense of miscategorizing 16.7% of benign cases, all malignant cases would have been correctly detected.

As stated in the Method section, we computed contrast ratio against both psoas muscle and myometrium as the presentation of outer myometrial layer could change during menstrual cycle [27] and psoas muscle might be a better reference for calculating the contrast. As shown in Table 4, the overall accuracy of tumor psoas contrast ratio on T2 was higher than that of tumor myometrium contrast ratio on T2. Using the McNemar test, a *p*-value of 0.027 was obtained and hence the difference between two accuracies was significant.

As stated in the Method section, we calculated the tumor-psoas and tumor-myometrium contrast ratios on CE-MRI image as well. As shown in Table 4, the overall accuracy of tumor psoas contrast ratio on T2 was higher than that of tumor myometrium contrast ratio on T2. Using the McNemar test, a *p*-value of 0.027 was obtained, hence the difference between the two accuracies was significant. The accuracy is slightly higher for the tumor myometrium contrast ratio on CE-MRI images, but the p-value obtained from the McNemar test was not significant (*p* = 0.572). On the other hand, between two tumor psoas contrast ratios, the one extracted from T2 images obtained a significantly higher accuracy (*p* = 0.029).

**Fig. 5:**
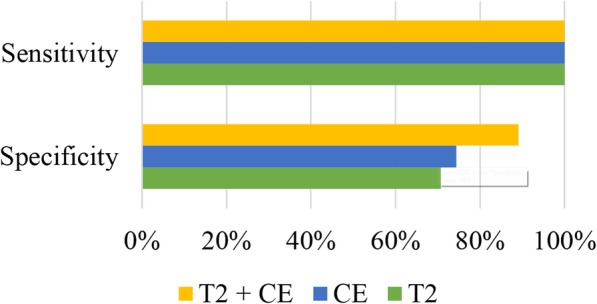
Sensitivity and specificity of the considered classifiers. Classifiers were relying on tumor myometrium contrast ratio on T2 images, tumor myometrium contrast ratio on CE images, and tumor myometrium contrast ratio on both T2 and CE images for distinguishing malignant masses from benign ones

We also investigated the benefit of combining tumor myometrium contrast ratio extracted from CE-MRI images with the similar figure extracted from T2-weighted sequences. To do so, we designed a classifier that classified a lesion as a malignancy only if tumor myometrium contrast ratios on both CE-MRI and T2 images were above the cut-off values shown in Table 4. Figure 5 shows the sensitivity and specificity of two original classifiers (presented in Table 4) in comparison with those of this new classifier. As shown, without any loss in sensitivity, the specificity increased by more than 15%. The McNemar test showed that the obtained accuracy for the new classifier based on the combination of two metrics differed significantly from that of the two original classifiers (both *p*-values< 0.001).

## Discussion

Previous studies suggested that the MRI has potential application in the differentiation of uterine sarcomas from leiomyomas. More specifically, it was shown that on T2-weighted images, the leiomyomas are well-circumscribed masses with a low signal intensity while sarcomas exhibit high-intensity spots [26, 30, 31]. However, degenerated leiomyomas could also appear as hypersignal lesions on T1-and T2-weighted images [14, 16–20, 32]. Therefore, signal intensity alone is not sufficient for the differentiation of benign lesions from malignant ones, especially in atypical leiomyomas with extensive necrosis or those with cystic or fibrinoid degeneration. Assessing marked hypercellularity or signs of neoangiogenesis are essential for making diagnosis. Perfusion and diffusion imaging can assess these features, however, they cannot be acquired for all patients and they are not part of standard MRI protocol, conducted for patients. Here, we investigated the discriminative power of the quantitative parameters other than those usually assessed by radiologists (e.g. signal intensity) for differentiating uterine sarcomas from leiomyomas. We included six quantitative metrics—T2 mapping parameter, T2 scaled ratio, tumor myometrium contrast ratio on T2, tumor psoas contrast ratio on T2, tumor myometrium contrast-enhanced ratio, and tumor psoas contrast-enhanced ratio — and showed that these metrics have promising discriminative power in the differentiation of uterine sarcomas from leiomyomas. To the best of our knowledge, these quantitative features have not been used previously for benign/malignant classification of myometrial masses.

We also calculated different diagnostic measures to assess the performance of the metrics in the binary classification of the masses. The results indicated that the T2 map reached the highest overall accuracy for categorizing the masses as benign or malignant. Meanwhile, the T2 scaled ratio, tumor myometrium contrast ratio on T2, and tumor myometrium contrast enhanced ratio achieved a sensitivity of 100%. This suggests that, if any of these metrics had been utilized as a tool for the benign/malignant categorization of uterine masses, none of the uterine sarcomas would have been misclassified as a benign mass. Among these three metrics, the T2 scaled ratio obtained the highest specificity. Based on this metric, with no loss of sensitivity, 83.3% of the benign masses, which could be treated less aggressively, were detected.

Our results demonstrated that the T2 map achieved the highest AUC value. This finding was in line with previous studies that highlighted its importance for differentiating benign lesions from malignancies in ovarian [23], breast [24], and prostate [33] tissue. Carter et al. showed that quantitative metrics from the CE-MRI and T2 mapping can distinguish benign from malignant ovarian masses [23]. In another study, the T2 mapping was found to be useful in differentiating benign breast masses from malignant ones [24]. Our findings demonstrated similar difference between benign and malignant uterine masses.

Our study also demonstrated that tumor myometrium contrast ratio calculated on CE-MRI images provides complementary information to the similar contrast ratio extracted from T2-weighted images. Our data showed that, by combining these two metrics, the specificity increased by more than 15% while a sensitivity of 100% was achieved. This promising result was achieved by using an equilibrium phase CE-MRI image. Also, the results suggested that tumor myometrium contrast ratios on CE-MRI and T2 images are sufficient to achieve a sensitivity of 100% and contrast ratios based on psoas muscle are not required.

Our study has a number of limitations. First, although the results obtained are promising, this could be due to the small sample size and lack of borderline cases in our dataset. A future step could be conducting a prospective study with a larger sample size. Second, we only included patients who underwent hysterectomy. Therefore, our dataset was skewed and did not represent the general population. Third, our data suggested that no differences in signal intensity on T1-wighted sequences existed between the two groups. In some previous studies, however, differences in T1 signal intensity have been observed between benign and malignant tumors [15, 16, 30]. This could be due to the limited number of patients included either in our study or in previous studies. Moreover, the value of this parameter, signal intensity on T2 sequences and the presence of central necrosis were assessed visually by a radiologist. Although she was an experienced practitioner, inter-observer variability should be evaluated. A future work for this study could be investigating the inter-radiologist agreement in assessing these parameters. We included all types of uterine sarcoma (carcinosarcoma, leiomyosarcoma, endometrial stromal sarcoma) in one category and did not evaluate imaging parameters of them separately and in comparison to each other. Nor did we include subtypes of leiomyomas such as degenerated, atypical, or cellular leiomyomas separately. In addition, outer myometrial layer could change during menstrual cycle [27] and patients in two cohorts should be comparable with regard to menstruation phase.

Previous studies showed efficacy of diffusion imaging [34] and perfusion-weighted MRI parameters [35] in distinguishing uterine sarcoma from leiomyomas. This study showed the quantitative T2-based metrics can be used in preoperative assessment of myometrial masses. Therefore, one possible future direction for this study could be combining the quantitative T2 parameters with features extracated from diffusion and perfusion imaging to build a high predictive classifier for differentiating benign leiomyomas from malignant uterine sarcomas.

## Conclusion

In summary, this study showed that the extracted quantitative metrics could be utilized in the preoperative assessment of uterine masses to differentiate benign leiomyomas from malignant uterine sarcomas. Our data suggested that tumor myometrium contrast ratio calculated on CE-MRI images provides complementary information to the similar contrast ratio extracted from T2-weighted images. We also proposed a novel framework, which combined these two metrics, for distinguishing leiomyomas from uterine sarcomas. Our framework achieved a sensitivity of 100% and a specificity of 89%. Therefore, by combining the T2-weighted image with an equilibrium phase CE-MRI image we can correctly detect 89% of benign lesions without any loss in sensitivity. This promising result was has major implications for improving current practice in the management of uterine masses through the non-invasive detection of benign cases that could be treated less aggressively.

## Abbreviations

AUC: Area under receiver operating characteristic
CE-MRI: Contrast-enhanced Magnetic Resonance Imaging
MRI: Magnetic Resonance Imaging
NPV: Negative Predictive Value
PPV: Positive Predictive Value
ROC: Receiver Operating Characteristic
ROI: Region of interest
